# The Value of Hemodynamic Measurements or Cardiac MRI in the Follow-up of Patients With Idiopathic Pulmonary Arterial Hypertension

**DOI:** 10.1016/j.chest.2020.10.077

**Published:** 2020-11-14

**Authors:** Cathelijne Emma van der Bruggen, Martin Louis Handoko, Harm Jan Bogaard, Johannes Timotheus Marcus, Franciscus Petrus Theodorus Oosterveer, Lilian Jacoba Meijboom, Berend Eric Westerhof, Anton Vonk Noordegraaf, Frances S. de Man

**Affiliations:** a Pulmonary Medicine, Amsterdam Cardiovascular Sciences, Amsterdam UMC Amsterdam, Vrije Universiteit Amsterdam, Amsterdam, The Netherlands; b Cardiology, Amsterdam Cardiovascular Sciences, Amsterdam UMC Amsterdam, Vrije Universiteit Amsterdam, Amsterdam, The Netherlands; c Radiology and Nuclear Medicine, Amsterdam Cardiovascular Sciences, Amsterdam UMC Amsterdam, Vrije Universiteit Amsterdam, Amsterdam, The Netherlands; d Cardiovascular and Respiratory Physiology, Faculty of Science and Technology, Technical Medical Center, University of Twente, Enschede, The Netherlands

**Keywords:** imaging, pulmonary hypertension, right ventricle, 6MWD, 6-min walking distance, AIC, Akaike information criterion, CMR, cardiac MRI, CO, cardiac output, ERS/ESC, European Respiratory Society/European Society of Cardiology, iPAH, idiopathic pulmonary arterial hypertension, mPAP, mean pulmonary arterial pressure, NT-proBNP, N-terminal pro-brain natriuretic peptide, NYHA, New York Heart Association, PAH, pulmonary arterial hypertension, PAWP, pulmonary arterial wedge pressure, PVR, pulmonary vascular resistance, RAP, right atrial pressure, RHC, right heart catheterization, RV, right ventricle, RVEDV, right ventricular end-diastolic volume, RVEF, right ventricular ejection fraction, RVESV, right ventricular end-systolic volume, SVi, stroke volume index, Svo_2_, mixed venous oxygen saturation

## Abstract

**Background:**

Treatment of patients with pulmonary arterial hypertension (PAH) is conventionally based on functional plus invasive measurements obtained during right heart catheterization (RHC). Whether risk assessment during repeated measurements could also be performed on the basis of imaging parameters is unclear, as a direct comparison of strategies is lacking.

**Research Question:**

How does the predictive value of noninvasive parameters compare with that of invasive hemodynamic measurements 1 year after the diagnosis of idiopathic PAH?

**Study Design and Methods:**

One hundred and eighteen patients with idiopathic PAH who underwent RHC and cardiac MRI (CMR) were included in this study (median time between baseline evaluation and first parameter measures, 1.0 [0.8-1.2] years). Forty-four patients died or underwent lung transplantation. Forward Cox regression analyses were done to determine the best predictive functional, hemodynamic, and/or imaging model. Patients were classified as high risk if the event occurred < 5 years after diagnosis (n = 24), whereas patients without event were classified as low risk.

**Results:**

A prognostic model based on age, sex, and absolute values at follow-up of functional parameters (6-min walk distance) performed well (Akaike information criterion [AIC], 279; concordance, 0.67). Predictive models with only hemodynamic (right atrial pressure, mixed venous oxygen saturation; AIC, 322; concordance, 0.66) or imaging parameters (right ventricular ejection fraction; AIC, 331; concordance, 0.63) at 1 year of follow-up performed similarly. The predictive value improved when functional data were combined with either hemodynamic data (AIC, 268; concordance, 0.69) or imaging data (AIC, 273; concordance, 0.70). A model composed of functional, hemodynamic, and imaging data performed only marginally better (AIC, 266; concordance, 0.69). Finally, changes between baseline and 1-year follow-up were observed for multiple hemodynamic and CMR parameters; only a change in CMR parameters was of prognostic predictive value.

**Interpretation:**

At 1 year of follow-up, risk assessment based on CMR is at least equal to risk assessment based on RHC. In this study, only changes in CMR, but not hemodynamic parameters, were of prognostic predictive value during the first year of follow-up.

Pulmonary arterial hypertension (PAH) is a progressive condition, characterized by extensive pulmonary vascular remodeling, resulting in an ongoing rise in right ventricular (RV) pressure overload.^1^ To secure RV systolic function and thus oxygen supply to all organs, the right ventricle adapts via several compensatory mechanisms. However, ultimately these are insufficient and progression to RV dysfunction and failure remains inevitable, with a grim prognosis as a result.^2^

According to guidelines, right heart catheterization (RHC) is recommended to support the diagnosis of PAH. In addition, RHC should be considered to guide treatment decisions including adjustments in treatment regimen and/or referral to a transplantation center (class IIa recommendation).^3^ However, whether hemodynamic assessment during follow-up is preferred above noninvasive monitoring is currently unclear.

A risk stratification algorithm has been published in the European Respiratory Society/European Society of Cardiology (ERS/ESC) guidelines and has been validated by several groups.^4^, ^5^, ^6^ In addition, studies by Weatherald et al^7^ and Chin et al^8^ have demonstrated that after 4 months of follow-up, right atrial pressure (RAP), stroke volume index (RHC-SVi), and serum levels of N-terminal pro-brain natriuretic peptide (NT-proBNP) are independently associated with death or lung transplantation and are therefore important parameters to determine treatment response in patients with PAH.

Notwithstanding the unquestionable predictive value of invasive hemodynamic assessment, several studies have shown that noninvasive imaging by cardiac MRI (CMR) also has great predictive value at baseline.^7^^,^^9^, ^10^, ^11^, ^12^, ^13^ The noninvasive character of CMR may enable easier routine follow-up of patients with PAH, as it is more patient-friendly and carries minimal patient risks. As in our institute functional, hemodynamic, and imaging assessments are routinely combined during follow-up, we have a unique cohort to compare the predictive value of these various modalities of monitoring patients with idiopathic PAH. Therefore, the aim of this study was to compare the predictive value of CMR parameters with that of invasive hemodynamic measurements at 1-year follow-up.

## Methods

### Study Objectives

We retrospectively evaluated all patients with idiopathic PAH (iPAH) or hereditary PAH seen between March 2000 and September 2018 at the Amsterdam University Medical Center (location, VU University Medical Center, The Netherlands, a tertiary referral center for PAH). A diagnosis of idiopathic or hereditary PAH was made by a multidisciplinary pulmonary hypertension team after extensive clinical evaluation according to the ERS/ESC guidelines in the relevant time period.^3^ Because the repeated functional, hemodynamic, and imaging measurements were performed for clinical purposes, this study did not fall within the scope of the Medical Research Involving Human Subjects Act (confirmed by the Medical Ethics Review Committee of the VU University Medical Center, 2012.288).

### Functional Assessment

Functional assessment of the patients was performed by 6-min walk distance (6MWD), New York Heart Association (NYHA) classification, and NT-proBNP level.

### Hemodynamic Assessment by Right Heart Catheterization

RHC was performed with a balloon-tipped, flow-directed, 7.5F triple-lumen Swan-Ganz catheter (Edwards Lifesciences LLC). Cardiac output (CO) was measured by either the Fick method or thermodilution (23% direct Fick, 4% indirect Fick, and 73% thermodilution). Pulmonary vascular resistance (PVR) was calculated as follows: PVR = (mPAP – PAWP)/CO, where mPAP is mean pulmonary arterial pressure and PAWP is pulmonary arterial wedge pressure.

### Imaging by Cardiac MRI

CMR was performed on a 1.5T Avanto or Sonata scanner equipped with a six-element phased array coil (Siemens Medical Solutions). Image acquisition and postprocessing were performed as described previously.^14^^,^^15^ The RV ejection fraction (RVEF) was calculated according to the following formula: RVEF = (RVEDV – RVESV)/RVEDV × 100%, where RVESV is RV end-systolic volume and RVEDV is RV end-diastolic volume. In line with Mauritz et al,^16^ stroke volume was calculated on the basis of left ventricular volumes (left ventricular end-diastolic volume [LVEDV] minus left ventricular end-systolic volume [LVESV]).

### Statistical Analysis

Data are presented as mean ± SD or median (interquartile range), depending on distribution. Categorical variables are presented as absolute numbers and relative frequencies (percentage). No further analyses were computed on the missing data, neither data imputation was executed.

Average and median values of general characteristics of patients at baseline were calculated with R package tableone.^17^ Individual baseline and follow-up graphs were constructed with R package ggpubr.^18^ Differences between baseline and follow-up were tested by paired *t* test for continuous variables and by McNemar test for categorical variables, with the Bonferroni correction applied for multiple comparisons.

The predictive value of functional, hemodynamic, and imaging parameters was tested by univariate and multivariate Cox regression analyses, using R package survival.^19^ Forward Cox regression modeling was used to determine the best predictive functional, hemodynamic, or imaging model, using *P* < .1 as cutoff, to be included in the final model. To compare the predictive value of the various models, the concordance (C statistics) and Akaike information criterion (AIC) are provided. The Akaike information criterion provides a relative measure of the quality of the model and estimates the information lost by the number of parameters in the models and the strength of the predictive value. A low number means less information lost and could be interpreted as a better model. The concordance or C statistic provides a measure of the goodness of fit in survival models. A higher concordance value means that the model gives a better prediction for survival. Pairwise comparisons were performed and corrected for multiple comparisons with the Bonferroni correction. All graphs were generated with ggplot2.^20^

All tests were two-sided, and *P* < .05 was considered significant. Statistical analyses were performed with RStudio (version 1.1.463; RStudio, Inc.).

## Results

### Baseline Characteristics

**Figure 1:**
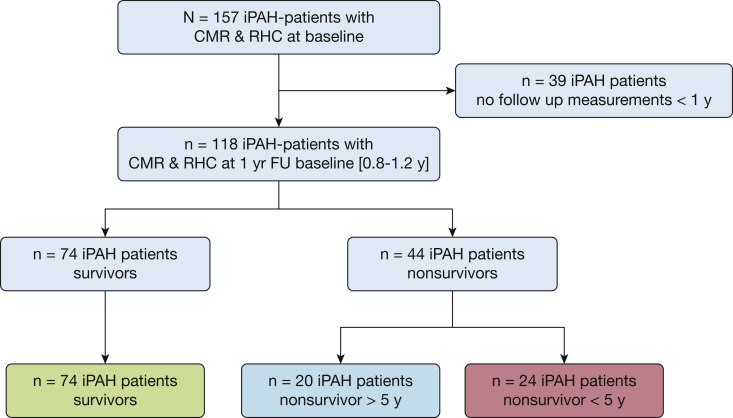
Flow chart of patient selection. In total, 157 patients with iPAH were identified who had CMR and RHC assessment at baseline. In 39 patients, no follow-up analyses including both CMR and RHC were available. Median follow-up time was 1 year with an interquartile range of 0.8 to 1.2 years. Of the 118 patients with follow-up measurements, two patients were excluded because from one patient no baseline data were available, and one patient was misclassified as having iPAH. In total, 44 patients died or underwent lung transplantation (24 patients had an event within 5 years of diagnosis, and 20 patients had an event more than 5 years after diagnosis). Patients without events were defined as survivors. CMR = cardiac MRI; FU = follow-up; iPAH = idiopathic pulmonary arterial hypertension; RHC = right heart catheterization.

As can be observed in Figure 1, baseline CMR and RHC data were available for 157 patients with iPAH. In 39 patients with iPAH, no complete follow-up with CMR and RHC was available and we therefore excluded these patients. In total, 118 patients with functional, hemodynamic, and imaging assessment at baseline and follow-up were included in this study with a median time between the baseline measurement and the following complete CMR + RHC measurement of 1.0 year and interquartile range of 0.8 to 1.2 years.

Sixty-three percent of the patients were classified as survivors, whereas in 37% an event (death or lung transplantation) occurred. In 24 patients this event occurred < 5 years after diagnosis. These patients were classified as high-risk patients, whereas patients classified as survivors were considered low-risk patients (Fig 1).

General characteristics of the patients are presented in Table 1. The study population was 49 ± 17 years old and consisted predominantly of women (69%). Most patients were in NYHA functional class II or III. In 52 of 118 patients (44%) monotherapy was the initial treatment strategy of choice. Combination therapy or triple therapy was initiated in, respectively, 51% and 5% of the study population.

**Table 1:** General Characteristics

Variable	Patients
General characteristics	
No. of patients	118
Age, y	49 ± 17
Sex, female, No. (%)	81 (69)
BMI	26 ± 4.9
Follow-up time, y	5.5 (3-8)
NYHA class (n = 106), No. (%)	
I	8 (7)
II	40 (35)
III	58 (51)
IV	7 (6)
Treatments during follow-up	
Monotherapy, No. (%)	52 (44)
Duotherapy, No. (%)	60 (51)
Triple therapy, No. (%)	6 (5)
Calcium antagonists, No. (%)	10 (8)
Endothelin antagonists, No. (%)	84 (71)
PDE5 inhibitors, No. (%)	76 (64)
Prostacyclin analog, No. (%)	18 (15)
Events	
No event > 5 y, No. (%)	42 (36)
No event < 5 y, No. (%)	32 (27)
Event > 5 y, No. (%)	20 (17)
Event < 5 y, No. (%)	24 (20)

Data presented as mean ± SD or median (interquartile range) when data are not normally distributed. NYHA = New York Heart Association; PDE5 = phosphodiesterase 5.

Patients presented with an mPAP of 54 ± 14 mm Hg, mixed venous oxygen saturation (Svo_2_) of 64% ± 8%, mRAP of 8 ± 5 mm Hg, NT-proBNP level of 657 (246-1,999) ng/L, and an RVEF of 36% ± 12%. The first follow-up was performed after a median time of 1.0 (0.8-1.2) years.

### Predictive Value of Functional, Hemodynamic, and Imaging Parameters

To determine the individual predictive value of functional, hemodynamic, and imaging parameters in our cohort, univariate Cox regression was performed. At follow-up, all variables except age were associated with the risk of death or lung transplantation (Table 2).

**Table 2:** Univariate Cox Regression Analyses of Functional, Hemodynamic, and Imaging Parameters

Parameter	Hazard Ratio	95% CI	*P* Value
Functional			
Age, y	1.02	1.00-1.04	.099
Sex, female vs male	0.41	0.22-0.76	< .001
6-min walk distance (per 10 m)	0.96	0.94-0.99	.002
NT-proBNP, pg/mL	1.20	1.04-1.39	.012
New York Heart Association (class I vs class II)	1.52	0.45-5.11	.500
New York Heart Association (class I vs class III)	5.38	1.55-18.74	.008
Hemodynamic			
RHC: Mean pulmonary artery pressure, mm Hg	1.02	1.00-1.04	.017
RHC: Stroke volume index, mL/m^2^	0.96	0.94-0.99	.003
RHC: Right atrial pressure, mm Hg	1.10	1.04-1.17	.002
RHC: Cardiac index, mL/m^2^	0.66	0.47-0.94	.020
RHC: Pulmonary vascular resistance index, dyn × s/cm^5^	1.69	1.10-2.60	.017
RHC: Mixed venous oxygen saturation, %	0.94	0.90-0.97	< .001
RHC: Heart rate, beats/min	1.02	0.99-1.04	.174
Imaging			
CMR: Stroke volume index, mL/m^2^	0.95	0.92-0.99	.008
CMR: RV ejection fraction, %	0.96	0.94-0.98	< .001
CMR: RV end-systolic volume index, mL/m^2^	1.02	1.01-1.03	< .001
CMR: RV end-diastolic volume index, mL/m^2^	1.02	1.00-1.03	.017

For sex, male patients are used as reference. CMR = cardiac MRI; NT-proBNP = N-terminal pro-brain natriuretic peptide; RHC = right heart catheterization.

A multivariate Cox regression analysis was performed to compare the additional value of CMR parameters. First, we determined the best functional model, using Cox regression analyses with age, sex, 6MWD, NYHA functional class, and NT-proBNP level. A model with only age, sex, and 6MWD reached statistical significance with an AIC of 279 and a concordance index of 0.67 (Table 3, e-Fig 1).

**Table 3:** Multivariate Cox Regression Analyses of Functional, Hemodynamic, and Imaging Parameters: Functional, Hemodynamic, and Imaging Models

Model	Parameter	Hazard Ratio	95% CI	*P* Value	AIC	Concordance
Model 1: Functional	Age	1.00	0.98-1.02	.681	279	0.67
	Sex	0.44	0.22-0.89	.023	…	…
	6MWD	1.00	0.99-1.00	.003	…	…
Model 2: Hemodynamic	Age	1.02	1.00-1.04	.062	322	0.66
	Sex	0.46	0.24-0.89	.021	…	…
	RHC-RAP	1.08	1.01-1.15	.024	…	…
	RHC-Svo_2_	0.94	0.90-0.98	.005	…	…
Model 3: Imaging	Age	1.03	1.01-1.05	.009	331	0.63
	Sex	0.52	0.27-0.99	.045	…	…
	CMR-RVEF	0.96	0.93-0.98	< .001	…	…

6MWD = 6-min walk distance; AIC = Akaike information criterion; CMR = cardiac MRI; RAP = right atrial pressure; RHC = right heart catheterization; RVEF = right ventricular ejection fraction; Svo_2_ = mixed venous oxygen saturation.

Subsequently, we determined the predictive value of the hemodynamic prognostic model, using forward Cox regression analyses with all hemodynamic parameters in addition to age and sex. Although the parameters individually all reached a significant predictive value, the forward Cox regression model resulted in a model containing age, sex, Svo_2_, and mRAP.

In a similar analysis for the CMR-derived right ventricular parameters, the forward Cox regression analyses resulted in a model containing age, sex, and RVEF.

The predictive values of the models with only hemodynamic or CMR parameters were comparable (Table 4). The combination of hemodynamic or CMR parameters with functional measurements provided the best predictive value. Combining all three modalities did result in a trivial improvement in quality of the prognostication (e-Fig 2). These analyses suggest that during follow-up, functional assessment in combination with either hemodynamic or imaging provides the best prognostic information.

**Table 4:** Multivariate Cox Regression Analyses of Functional, Hemodynamic, and Imaging Parameters: Functional, Hemodynamic, and Imaging Models Combined

Model	Parameter	Hazard Ratio	95% CI	*P* Value	AIC	Concordance
Model 1 + 2: Functional + hemodynamic	Age	1.01	0.98-1.01	.539	268	0.69
	Sex	0.43	0.21-0.89	.022	…	…
	6MWD	1.00	0.99-1.00	.220	…	…
	RHC-RAP	1.05	0.98-1.13	.180	…	…
	RHC-Svo_2_	0.95	0.90-1.00	.064	…	…
Model 1 + 3: Functional + imaging	Age	1.01	0.99-1.04	.298	273	0.70
	Sex	0.51	0.25-1.05	.069	…	…
	6MWD	1.00	0.99-1.00	.045	…	…
	CMR-RVEF	0.96	0.94-0.99	.004	…	…
Model 2 + 3: Hemodynamic + imaging	Age	1.03	1.01-1.05	.012	320	0.71
	Sex	0.49	0.26-0.95	.036	…	…
	RHC-RAP	1.05	0.98-1.12	.180	…	…
	RHC-Svo_2_	0.96	0.92-1.01	.102	…	…
	CMR-RVEF	0.97	0.94-1.00	.040	…	…
Model 1 + 2 + 3: Functional + hemodynamic + imaging	Age	1.02	0.99-1.04	.227	266	0.69
	Sex	0.47	0.23-0.98	.044	…	…
	6MWD	1.00	0.99-1.00	.220	…	…
	RHC-RAP	1.02	0.95-1.10	.577	…	…
	RHC-Svo_2_	0.98	0.92-1.04	.451	…	…
	CMR-RVEF	0.97	0.94-1.00	.052	…	…

For sex, male patients are used as reference. 6MWD = 6-min walk distance; AIC = Akaike information criterion; CMR = cardiac MRI; RAP = right atrial pressure; RHC = right heart catheterization; RVEF = right ventricular ejection fraction; Svo_2_ = mixed venous oxygen saturation.

### Change in CMR Parameters Discriminates Between High- and Low-Risk Patients

**Figure 2:**
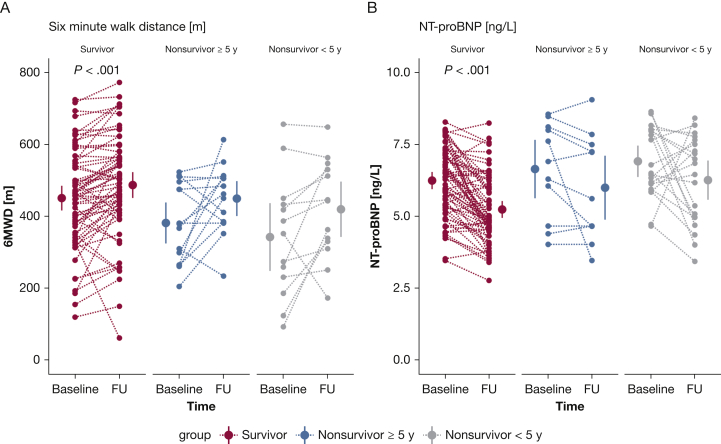
Treatment effect on functional parameters in patients with low- and high-risk profiles. A and B, A significant improvement in 6-min walk distance (A) and NT-proBNP (B) is observed only in patients with low risk (survivors). Nonsurvivor patients with short survivor time (< 5 years) are classified as high-risk patients, whereas survivor patients with follow-up data > 5 years after diagnosis were classified as low-risk patients. All *P* values are corrected for multiple comparisons (Bonferroni correction). 6MWD = 6-min walk distance; FU = follow-up; NT-proBNP = N-terminal pro-brain natriuretic peptide.

**Figure 3:**
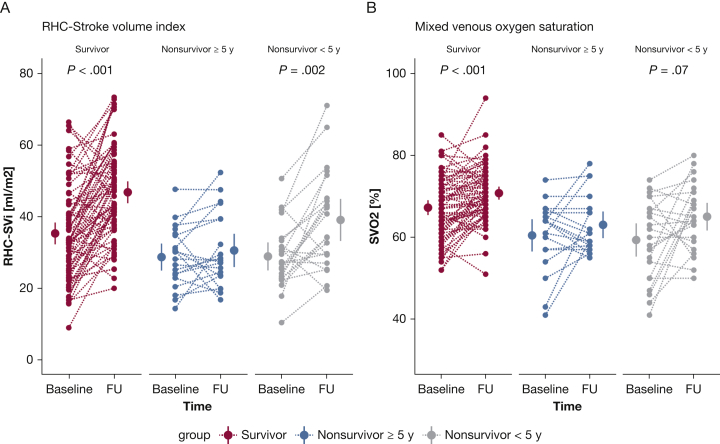
Treatment effect on hemodynamic parameters in patients with low- and high-risk profiles. Improvements in hemodynamic parameters are observed both in patients with low risk of mortality and patients with high risk of mortality (Table 5). A and B, Individual data on stroke volume index (A) and mixed venous oxygen saturation (B) are demonstrated at baseline and after 1 year of follow-up. All *P* values are corrected for multiple comparisons (Bonferroni correction). FU = follow-up; RHC = right heart catheterization; SVi = stroke volume index; Svo_2_ = mixed venous oxygen saturation.

**Figure 4:**
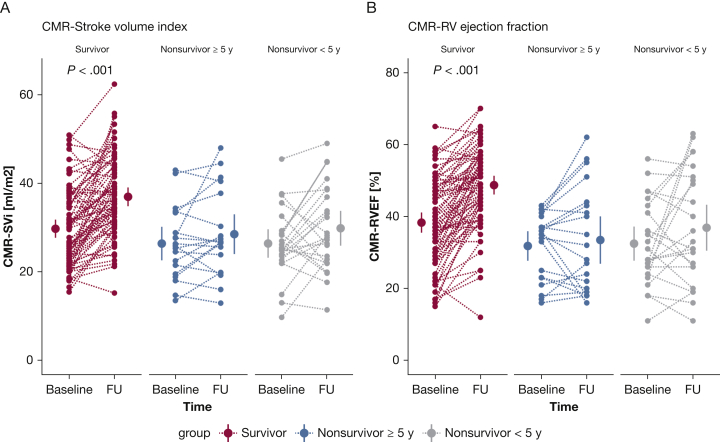
Treatment effect on CMR parameters in patients with low- and high-risk profiles. Significant improvements in RV function and morphology were observed only in patients with a low-risk profile. A and B, Individual data on stroke volume index, determined by CMR (A) and RV ejection fraction (B), are demonstrated. CMR = cardiac MRI; FU = follow-up; RVEF = right ventricular ejection fraction; SVi = stroke volume index.

To determine whether a change between baseline measurements and measurements after 1 year would provide further information on the clinical status of the patients, we assessed the discriminative value of the change in functional, hemodynamic, and imaging parameters to identify low- and high-risk patients (Table 5). High-risk patients were defined as patients having an event within 5 years of diagnosis. Low-risk patients had no event during > 5 years of follow-up. Treatment and comorbidities in the distinctive groups can be observed in the online article (e-Tables 1 and 2). Because of the arbitrary nature of the 5-year cutoff, analyses were repeated with a cutoff of an event within 3 and 7 years after diagnosis, as can be observed in the online article (e-Tables 3 and 4). Hemodynamic parameters showed improvements in all groups at follow-up independent of high- or low-risk patients (Fig 3). In contrast, 6MWD and NT-proBNP (Fig 2) and RV function (Fig 4, e-Fig 2) improved only in low-risk patients. To further corroborate this finding, we determined the prognostic value of a change in functional, hemodynamic, and imaging parameters. Only a change in RVEF was significantly associated with survival after correction for possible confounding by sex and age (hazard ratio, 0.96; 95% CI, 0.92-0.99; *P* = .005). This suggests that although a patient improves hemodynamically, this does not necessarily translate into improved prognosis.

**Table 5:** Patient Hemodynamic, Functional, and Right Ventricular Characteristics at Baseline and 1-Year Follow-up, Stratified on Survival Time

	Survivors (N = 74)		Nonsurvivors			
			≥ 5 Years (n = 20)		< 5 Years (n = 24)	
	Baseline	FU	Baseline	FU	Baseline	FU
Functional						
Age, y	48 ± 17		47 ± 14		54 ± 20	
Sex, female, %	86		70		54	
6MWD, m	450 ± 17	493 ± 18^b^	382 ± 28	449 ± 24	345 ± 44	418 ± 36
NT-proBNP, ng/L	586 [215-1,292]	138 [94-442]^b^	997 [266-3,136]	274 [106-1,597]	775 [439-2,950]	785 [143-1,881]
NYHA I/II/III/IV, %	8/44/46/3	26/67/8/0^a^	5/35/55/5	10/75/15/0	5/15/70/10	5/35/60/0
Hemodynamic						
RHC-SVi, mL/m^2^	35 ± 2	47 ± 2^b^	29 ± 2	31 ± 2	29 ± 2	38 ± 3^b^
RAP, mm Hg	7 ± 0.4	5 ± 0.4^a^	10 ± 1.4	9 ± 1.1	10 ± 1.2	8 ± 1.2
RHC-CI, L/min/m^2^	2.7 ± 0.1	3.4 ± 0.1^b^	2.2 ± 0.1	2.5 ± 0.2	2.5 ± 0.2	3.2 ± 0.3^c^
RHC-PVRI, WU/m^2^	4.9 ± 0.3	2.7 ± 0.2^b^	5.7 ± 1.4	3.6 ± 1.0^a^	6.7 ± 0.8	4.1 ± 0.7^b^
RHC-Svo_2_, %	67 ± 1	71 ± 1^b^	61 ± 2	63 ± 2	59 ± 2	65 ± 2^a^
RHC-HR, beats/min	79 ± 2	75 ± 1^a^	79 ± 4	83 ± 3	89 ± 4	80 ± 3^a^
Imaging						
CMR-SVi, mL/m^2^	30 ± 1	37 ± 1^b^	26 ± 2	29 ± 2	26 ± 2	30 ± 2
CMR-RVEF, %	38 ± 1	49 ± 1^b^	32 ± 2	33 ± 3	32 ± 2	37 ± 3
CMR-RVESVi, mL/m^2^	49 ± 2	40 ± 2^b^	59 ± 5	58 ± 5	55 ± 4	55 ± 5
CMR-RVEDVi, mL/m^2^	77 ± 2	76 ± 2	85 ± 5	84 ± 5	80 ± 4	84 ± 5

All comparisons are between baseline and follow-up measurements. *P* values are corrected for multiple comparisons. 6MWD = 6-min walk distance; CI = cardiac index; FU = follow-up; HR = heart rate; NT-proBNP = N-terminal pro-brain natriuretic peptide; NYHA = New York Heart Association; PVRi = pulmonary vascular resistance index; RAP = right atrial pressure; RHC = right heart catheterization; RVEDVi = right ventricular end-diastolic volume index; RVEF = right ventricular ejection fraction; RVESVi = right ventricular end-systolic volume index; SVi = stroke volume index; Svo_2_ = mixed venous oxygen saturation; WU = Wood unit.

a *P* < .05.

b *P* < .001.

c *P* < .01.

## Discussion

In the present study, we compared the predictive values of functional, hemodynamic, and imaging parameters 1 year after the diagnosis of iPAH. We were able to demonstrate the following:

1. Both invasive RHC and CMR parameters have prognostic value at follow-up.
2. The predictive values of follow-up with RHC (RHC-Svo_2_ and mRAP) and CMR (RVEF) alone were similar.
3. The predictive value of a model combining functional parameters with hemodynamic or imaging parameters provided the best predictive model. Combining all three modalities did not provide further prognostic information. The additional values of hemodynamic or imaging parameters combined with functional parameters were similar.
4. With repeated measurements after 1 year of follow-up, hemodynamic parameters improved even in nonsurvivors, whereas RV imaging and functional parameters improved only in survivors. Only a change in RVEF was shown to be of predictive value.

Taking these findings together, we can conclude that follow-up by CMR is at least equal to invasive hemodynamic measurements to discriminate between high- and low-risk patients with PAH, and that combining functional with either hemodynamic or imaging measurements is of additional value during the follow-up of patients with PAH.

### Predictive Value of Noninvasive Follow-up Using CMR

RHC is recommended in the ERS/ESC guidelines at the time of diagnosis. In addition, there is a class IIa recommendation to perform RHC to assess treatment response and in case of clinical deterioration.^3^ However, as is known from multiple registry studies, not all PAH institutes perform routine hemodynamic assessment at follow-up. It can be argued that an invasive follow-up strategy is not superior to a noninvasive strategy, as a direct comparison is currently lacking. In this unique study we were able to compare three follow-up strategies: (1) functional: with 6MWD, NT-proBNP, and NYHA functional class; (2) hemodynamic: with RHC-obtained data; and (3) imaging: with CMR-derived data. The best predictive model was obtained by combining functional assessment with imaging and hemodynamic analyses. However, the differences between the models containing functional assessment with imaging or hemodynamics were minimal. This indicates that routine follow-up of patients with iPAH may be performed noninvasively, using CMR.

Recent advances with artificial intelligence in cardiovascular imaging may open new opportunities to combine multiple clinical modalities and CMR in the future.^21^ Future prospective cohort studies should reveal whether such an approach is beneficial and should be incorporated in the treatment algorithm of PAH.

### Changes in Invasive and Noninvasive Assessments After 1 Year of Follow-Up

Noninvasive imaging was performed by CMR, because previous studies have shown that CMR is more sensitive in detecting changes in RV function over time than is echocardiography.^22^ After 1 year of follow-up, no change in RAP and RVEDVi was observed, whereas only patients with a low risk of mortality (not those at high risk) showed significant improvements in 6MWD, NT-proBNP, CMR-SVi, RVEF, and RVESVi. In contrast, improvement in hemodynamic parameters was observed in low- and high-risk patients (nonsurvivors < 5 years). This indicates that patients can still be at high risk for mortality although their hemodynamic parameters improved.

### Right Ventricle as a Treatment Target

The results of this study highlight again the importance of RV adaptation on prognosis of patients with iPAH. All identified predictive markers are closely related to reduced RV function. Unfortunately, to date no specific RV therapies exist.^2^ Current pharmaceutic strategies are not sufficient to normalize RV function.^15^ This is confirmed in our study, in which no improvements in RV volume or RAP could be observed after 1 year of treatment. More recent studies have suggested that when PVR is reduced by > 40%, reverse remodeling of the right ventricle may occur.^23^^,^^24^ Future treatment strategies with (upfront) double or triple therapy^25^^,^^26^ may prove to induce early reverse remodeling of the right ventricle and ultimately may improve the prognosis of patients with this serious condition. Another possible way to improve the prognosis of patients with PAH is to identify novel therapeutic compounds directed to the right ventricle. However, it is difficult to identify proper treatment targets when tissue is available only in end-stage right heart failure or from animal models with important differences in RV physiology when compared with humans.^27^

### Study Limitations

The sample size of this study is relatively small in comparison with earlier studies, due to the strict selection criteria. This limited the possibilities to perform multivariate regression analyses including general prognostic markers of PAH. In addition, the relatively small sample size might have led to an overfit of the developed models, which should be validated in larger populations.

More importantly, this is the first study in which paired analyses of functional, hemodynamic, and imaging parameters are performed in a sample size of 118 patients with iPAH with a meaningful follow-up of more than 5 years.

Because prognostic importance was determined after 1 year of follow-up, there may have been a selection bias by not selecting patients who died or received a lung transplant in the first year after diagnosis. In addition, because of the retrospective nature of this study, we had missing values in hemodynamic and functional parameters.

This study was performed in a single center. Although the overall hemodynamic and general characteristics of our iPAH population were comparable with previous published studies by Weatherald et al,^7^ our 5-year survival was higher. This may be explained by the difference in mean age between both studies (average age in our study, 49 years; average age in Weatherald et al study, 64 years). Age has been shown previously to be a key prognostic indicator in PAH.^5^ The age difference between the studies can further be explained by the high prevalence of patients with hereditary PAH included in this study (20%), who are on average diagnosed at an earlier age than patients with iPAH.^28^

The definition of high vs low risk at 5 years of follow-up is arbitrary. Similar results were obtained when repeating the analyses with a cutoff of 3 and 7 years (e-Tables 1 and 2), which demonstrates the robustness of our analysis.

## Interpretation

Risk assessment based on CMR measurements is at least equal to risk assessment based on invasive hemodynamic measurements during repeated measurements 1 year after diagnosis.

**Take-home Point**

Risk assessment based on CMR measurements is at least equal to risk assessment based on invasive hemodynamic measurements during repeated measurements 1 year after diagnosis.

## Supplementary Data

e-Online Data
