# Supplementary material for: The Value of Hemodynamic Measurements or Cardiac MRI in the Follow-up of Patients With Idiopathic Pulmonary Arterial Hypertension
Source: Chest. 2020 Nov 14;159(4):1575–85. doi: 10.1016/j.chest.2020.10.077 (PMC8039009; doi:10.1016/j.chest.2020.10.077)
Supplement: e-Online Data [file mmc1.pdf]

CHEST Online Supplement

The Value of Hemodynamic Measurements or Cardiac MRI in the Follow-up of Patients With Idiopathic Pulmonary Arterial Hypertension

Cathelijne Emma van der Bruggen, MD; Martin Louis Handoko, MD, PhD; Harm Jan Bogaard, MD, PhD; Johannes Timotheus Marcus, PhD; Franciscus Petrus Theodorus Oosterveer; Lilian Jacoba Meijboom, MD, PhD; Berend Eric Westerhof, PhD; Anton Vonk Noordegraaf, MD, PhD; and Frances S. de Man, PhD

CHEST 2021; 159(4):1575-1585

Online supplements are not copyedited prior to posting and the author(s) take full responsibility for the accuracy of all data.

© 2020 AMERICAN COLLEGE OF CHEST PHYSICIANS. Reproduction of this article is prohibited without written permission from the American College of Chest Physicians. See online for more details. **DOI:** 10.1016/j.chest.2020.10.077

e-Table 1 – Treatment, stratified on event time of 5 years

	Survivor	Non-Survivor		
	n=74	≥5 year (n=20)	<5 year (n=24)	P-value
Monotherapy	26 (35%)	13 (65%)	13 (54%)	0.01
Duotherapy	45 (61%)	6 (30%)	8 (33%)	
Triple therapy	2 (3%)	0 (0%)	3 (13%)	
Calcium-antagonist	6 (8%)	3 (15%)	1 (4%)	ns
Endothelin receptor antagonist	58 (78%)	13 (65%)	13 (54%)	0.04
PDE-5 inhibitor	52 (70%)	5 (25%)	19 (79%)	<0.001
Prostacyclin analogue	6 (8%)	7 (35%)	5 (21%)	<0.01

e-Table 2 – Comorbidities, stratified on event time of 5 years

	Survivor	Non-Survivor		
	n=74	≥5 year (n=20)	<5 year (n=24)	P-value
Coronary disease	3 (4%)	0 (0%)	3 (12.5%)	0.157
Hypertension	12 (16%)	1 (5%)	5 (20.8%)	0.380
Diabetes Mellitus	3 (4%)	2 (10%)	3 (12.5%)	0.197
Thyroid disease	7 (9%)	0 (0%)	1 (4.2%)	0.440
Pulmonary disease				
COPD Gold I-II	1 (1%)	1 (5%)	3 (12.5%)	0.041
OSAS	3 (4%)	1 (5%)	1 (4.2%)	1.000
Asthma	7 (9%)	1 (5%)	2 (8.4%)	1.000
Malignancy	7 (9%)	1 (5%)	1 (4.2%)	0.637

Online supplements are not copyedited prior to posting and the author(s) take full responsibility for the accuracy of all data.

e-Table 3 – Patients hemodynamic and imaging characteristics at baseline and 1 year follow-up stratified on event time of 3 years

		Survivor		Non-survivor			
		N=74		≥ 3 years N=39		< 3 years N=15	
		Baseline	FU	Baseline	FU	Baseline	FU
Functional	Age (years)	48 ± 17		50 ± 18		52 ± 19	
	Sex (% female)	86		66		53	
	6 MWD (m)	450 ± 17	493 ± 18***	379 ± 28	441 ± 22	330 ± 58	423 ± 51
	NTproBNP (ng/L)	586 [215-1292]	138 [94-442] ****	563 [350-2679]	634 [106-1613]	2518 [619-3155]	575[143-1946]
	NYHA I/II/III/IV (%)	8/44/46/3	26/67/8/0*	7/29/61/4	7/71/21/0	0/17/67/17	8/17/75/0
Hemodynamic	RHC-SVi (ml/m²)	35 ± 2	47 ± 2****	29 ± 1	32 ± 2	28 ± 3	41 ± 4**
	RAP (mmHg)	7 ± 0.4	5 ± 0.4*	9 ± 1	8 ± 1	11 ± 2	8 ± 2
	RHC-CI (L/min/m²)	2.7 ± 0.1	3.4 ± 0.1****	2.3 ± 0.1	2.6 ± 0.2	2.4 ± 0.2	3.3 ± 0.4
	RHC-PVRi (WU/m²)	4.9 ± 0.3	2.7 ± 0.2****	6.1 ± 1.1	4.2 ± 0.9	6.6 ± 0.7	3.8 ± 0.7**
	RHC-SVO_2 (%)	67 ± 1	71 ± 1****	62 ± 2	63 ± 1	57 ± 3	65 ± 2
	RHC-HR (beats/min)	79 ± 2	75 ± 1*	81 ± 3	81 ± 3	87 ± 5	81 ± 4
Imaging	CMR-SVi (ml/m²)	30 ± 1	37 ± 1****	27 ± 1	28 ± 2	26 ± 2	31 ± 3
	CMR-RVEF (%)	38 ± 1	49 ± 1****	32 ± 2	35 ± 3	32 ± 3	37 ± 4
	CMR-RVESVi (ml/m²)	49 ± 2	40 ± 2****	57 ± 4	55 ± 4	56 ± 6	58 ± 7
	CMR-RVEDVi (ml/m²)	77 ± 2	76 ± 2	83 ± 4	81 ± 4	81 ± 6	90 ± 6

Online supplements are not copyedited prior to posting and the author(s) take full responsibility for the accuracy of all data.

CHEST™ Online Supplement

e-Table 4 - Patients hemodynamic and imaging characteristics at baseline and 1 year follow-up stratified on event time of 7 year

		Survivor		Non-survivor			
		N=74		≥ 7 years N=14		< 7 years N=30	
		Baseline	FU	Baseline	FU	Baseline	FU
Functional	Age (years)	48 ± 17		44 ± 14		54 ± 19	
	Sex (% female) - *	86		71		57	
	6 MWD (m)	450 ± 17	493 ± 18***	383 ± 39	419 ± 32	354 ± 35	443 ± 29*
	NTproBNP (ng/L)	586 [215-1292]	138 [94-442] ****	769 [455-1766]	190 [105-1097]	887 [380-3044]	936 [143-1807]
	NYHA I/II/III/IV (%)	8/44/46/3	26/67/8/0*	7/43/43/7	0/79/21/0	4/15/73/8	12/42/46/0
Hemodynamic	RHC-SVi (ml/m²)	35 ± 2	47 ± 2****	29 ± 2	29 ± 2	29 ± 2	38 ± 3***
	RAP (mmHg)	7 ± 0.4	5 ± 0.4*	11 ± 2	9 ± 1	10 ± 1	8 ± 1
	RHC-CI (L/min/m²)	2.7 ± 0.1	3.4 ± 0.1****	2.1 ± 0.1	2.3 ± 0.2	2.4 ± 0.1	3.1 ± 0.2*
	RHC-PVR (WU/m²)	4.9 ± 0.3	2.7 ± 0.2****	6.7 ± 2.7	3.8 ± 1.7	6.3 ± 0.7	4.0 ± 0.6**
	RHC-SVO$_2$ (%)	67 ± 1	71 ± 1****	63 ± 2	64 ± 2	59 ± 2	64 ± 1*
	RHC-HR (beats/min)	79 ± 2	75 ± 1*	74 ± 4	81 ± 4	88 ± 3	81 ± 3
Imaging	CMR-SVi (ml/m²)	30 ± 1	37 ± 1****	26 ± 2	28 ± 3	27 ± 1	30 ± 2
	CMR-RVEF (%)	38 ± 1	49 ± 1****	32 ± 2	34 ± 4	32 ± 2	36 ± 3
	CMR-RVESVi (ml/m²)	49 ± 2	40 ± 2****	56 ± 6	53 ± 6	57 ± 4	58 ± 5
	CMR-RVEDVi (ml/m²)	77 ± 2	76 ± 2	82 ± 6	79 ± 6	82 ± 4	87 ± 4

Online supplements are not copyedited prior to posting and the author(s) take full responsibility for the accuracy of all data.

e-Figure 1 – Treatment effect on imaging parameters in patients with low-, and high-risk profiles

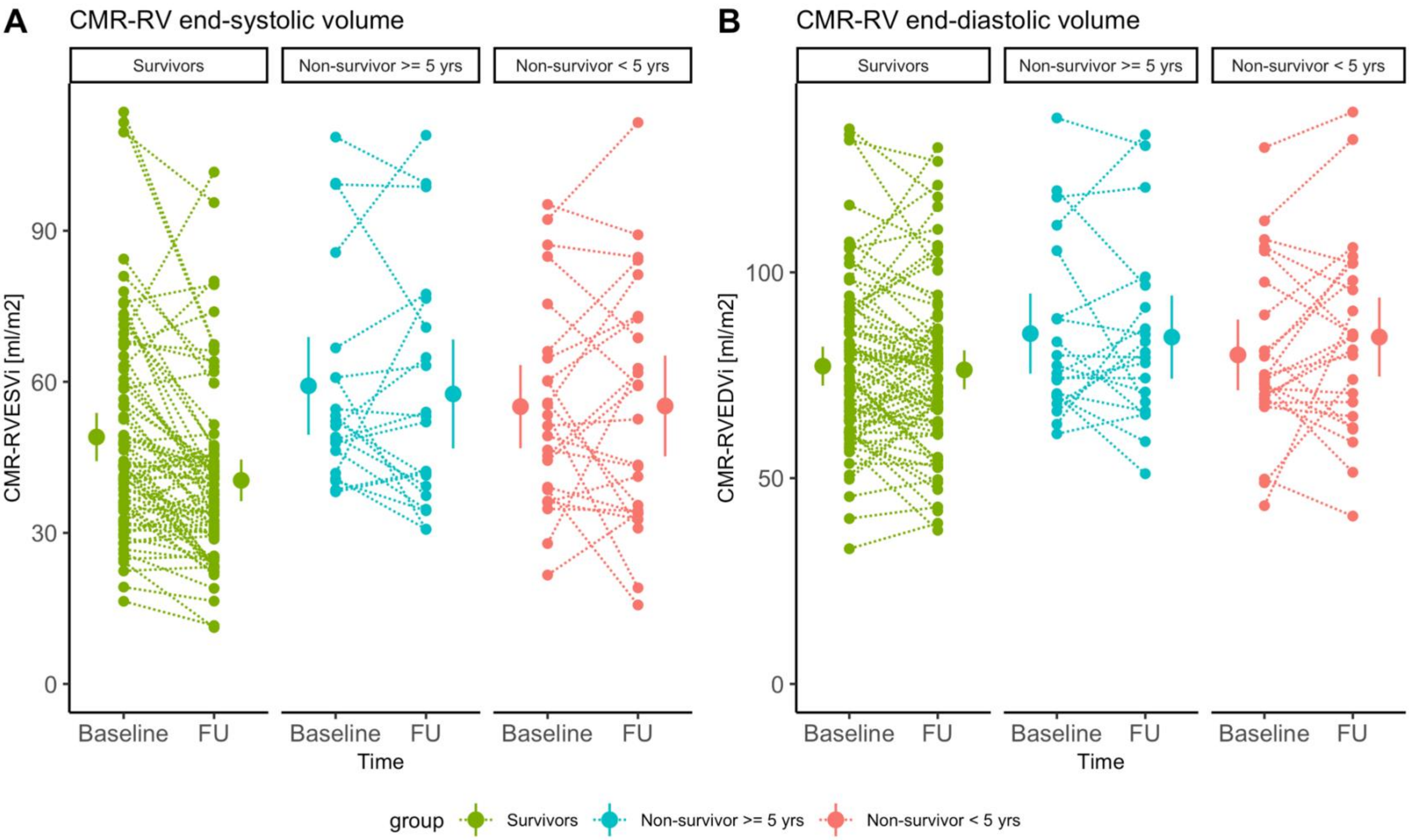

Online supplements are not copyedited prior to posting and the author(s) take full responsibility for the accuracy of all data.

e-Figure 2 – Akaike information criterion and concordance index of the predictive models

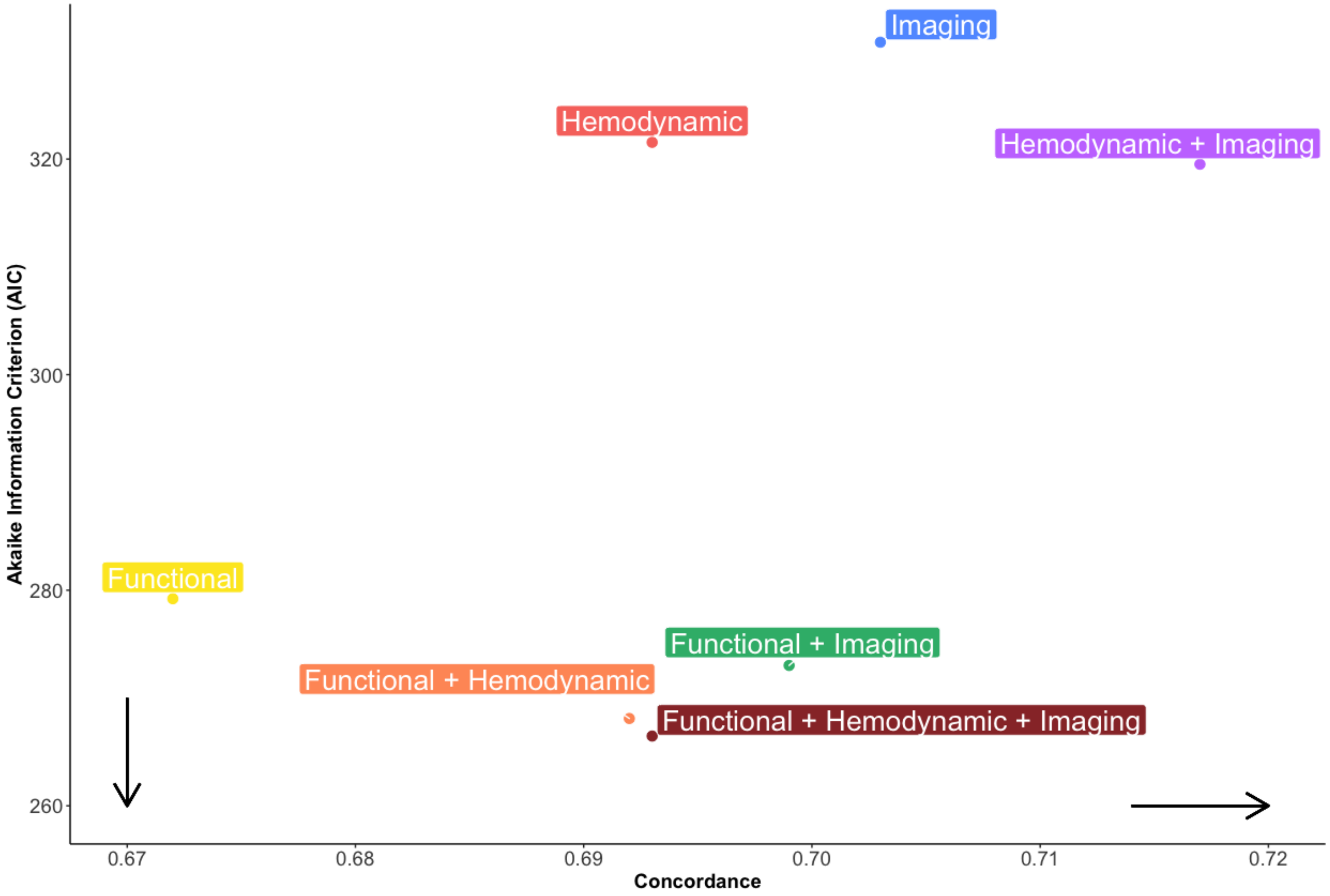

The Akaike information criterion provides a relative measure of the quality of the model and estimates the information lost by the number of parameters in the models and the strength of the predictive value. A low number means less information lost and could be interpreted as a better model. The concordance or C-statistics provides measure of goodness-of-fit in survival models. A higher concordance value means that the model gives a better prediction for survival. All models constructed by forward cox regression analyses are presented: Model 1 – Functional: Age + Sex + 6MWD; Model 2 – Hemodynamic: Age + Sex + RHC-mRAP + RHC-SvO_2; Model 3 – Imaging: Age + Sex + RVEF; Model 1+2: Age + Sex + 6MWD + RHC-mRAP + SvO_2, Model 1+3: Age + Sex + 6MWD + CMR-RVEF; Model 2+3: Age + Sex + RHC- mRAP + RHC-SvO_2 + RVEF; Model 1+2+3: Age + Sex + 6MWD + RHC-mRAP + RHC-SvO_2 + CMR-RVEF. The combination of functional with either hemodynamic and/or imaging parameters yielded highest predictive value.
Abbreviations: NYHA, New York Heart Association class; 6MWD, 6-minute walk distance; RHC, right heart catheterization; SVi, stroke volume index; RAP, right atrial pressure; SVO_2, mixed venous oxygen saturation; CI, cardiac index; CMR, cardiac magnetic resonance imaging, RVEDVi, RV end-diastolic volume index.

Online supplements are not copyedited prior to posting and the author(s) take full responsibility for the accuracy of all data.
